# Cystic fibrosis modulator therapy can reverse cystic bronchiectasis

**DOI:** 10.1002/rcr2.1172

**Published:** 2023-06-12

**Authors:** Peter G. Middleton, Nicholas J. Simmonds

**Affiliations:** ^1^ CF Service, Department Respiratory & Sleep Medicine Westmead Hospital Sydney New South Wales Australia; ^2^ Westmead Clinical School University of Sydney Sydney New South Wales Australia; ^3^ Adult Cystic Fibrosis Centre Royal Brompton Hospital London UK; ^4^ Respiratory Medicine Imperial College London, Royal Brompton Hospital London UK

**Keywords:** bronchiectasis, cystic fibrosis, elexacaftor, ivacaftor, tezacaftor

## Abstract

Bronchiectasis is often considered progressive and irreversible, so cases of regression or reversal are an important step in understanding the underlying pathophysiological mechanisms. Cystic fibrosis, (CF) caused by pathogenic variants in the *cystic fibrosis transmembrane conductance regulator* (*CFTR*) gene has been a success story in personalized medicine. The recent development of *CFTR* modulator therapies has revolutionized care. Dramatic improvements in lung function, sputum production, daytime functioning, and quality of life are seen within weeks. However, the effect of long‐term exposure to elexacaftor + tezacaftor + ivacaftor (ETI) on the structural abnormalities is at present unknown. This case series outlines three adults with CF who have demonstrated progressive improvement in the cylindrical, varicose and importantly cystic changes of bronchiectasis with prolonged ETI treatment. This raises the exciting question of reversibility of bronchiectasis as well as the mechanisms involved in the maintenance and progression of bronchiectasis as it relates to CF.

## Graphical abstract

Bronchiectasis, previously described as permanent and progressive airway dilatation, ranges from cylindrical to varicose to cystic. This report describes the reversal of cystic bronchiectasis following a year of CF modulator therapy, providing exciting new insights into the pathogenesis of bronchiectasis in CF.

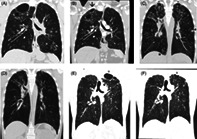

## INTRODUCTION

Bronchiectasis is described as the permanent dilatation of the airways on high‐resolution CT scan. Changes include cylindrical bronchiectasis, characterized by dilated airways that have lost their normal tapering, varicose bronchiectasis characterized by focal areas of constriction and dilation along the airway and cystic bronchiectasis, characterized by progressive dilatation of the airways which then end in large cysts or saccules, often described as the most severe form of bronchiectasis.^1^ Much of the literature concerning the aetiology and pathogenesis of bronchiectasis relates to the ‘vicious cycle’ as hypothesised by Cole more than 30 years ago, where inflammation and infection causes airway destruction, increased mucous retention and then further infection.^2^ It is frequently stated that ‘*Once the pattern is established, it becomes a progressive process over time*…’.^3^ Progressive destruction of the airways leads to worsening lung disease and ultimately respiratory compromise. More recently, the interactions between the primary cause, airway inflammation, airway infection and airway dysfunction have been expanded to the ‘vicious vortex’ where each individual component affects all the other components.^4^ But the fundamental tenet remains that once established, bronchiectasis progresses inexorably. In this way, finding cases of regression or reversal of bronchiectasis will be an important step in understanding the underlying pathophysiological mechanisms.

One of the well‐known causes of bronchiectasis is cystic fibrosis, (CF) caused by pathogenic variants in the *cystic fibrosis transmembrane conductance regulator* (CFTR) gene. The recent development of therapies to modulate CFTR in the majority of individuals with CF has revolutionized care with dramatic improvements in lung function, sputum production, daytime functioning, and quality of life.^5^, ^6^ It would be expected that CFTR modulators would improve bronchial wall thickening and mucous plugging in keeping with these clinical changes. This report outlines that long‐term elexacaftor + tezacaftor + ivacaftor (ETI) treatment can improve both these and the structural abnormalities of cystic bronchiectasis in three adults with F508del/F508del CF.

## CASE SERIES

**FIGURE 1:**
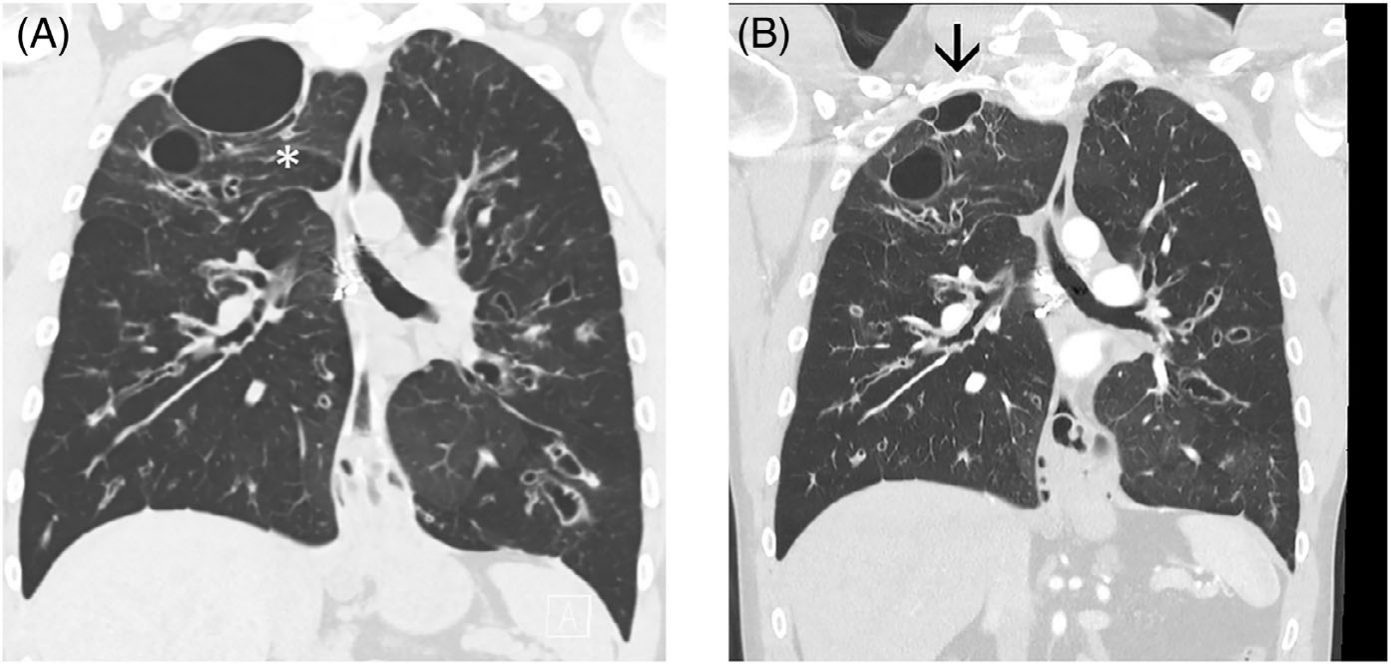
CT scans before (A) and after (B) elexacaftor + tezacaftor + ivacaftor for case 1

The first case, a 32‐year‐old male, with large cysts at the right apex on HRCT (Figure 1A) which had gradually increased in size: 2015 (29 × 15 × 21 mm), 2016 (50 × 33 × 30 mm) 2018 (56 × 44 × 31 mm) and 2020 (88 × 62 × 44mm). His FEV_1_/FVC was 1.11/2.71 (24%, 48% predicted) when he commenced ETI. After 6 months his spirometry had improved to 1.45/3.85 (31%, 68% predicted) and remained improved. A repeat CT scan after 18 months of therapy (Figure 1B) showed improvement in bronchial wall thickening, as expected. In addition, the size of the cystic and varicose airways decreased, with a dramatic reduction in the size of the large cyst at the right apex (61 × 45 × 22 mm), (arrow). The smaller cyst at the R apex also reduced in maximum dimensions, not shown on these cuts optimized for the dominant apical cyst. Interestingly, there appeared to also be a reduction in the compression of the surrounding lung from the first scan, shown with asterisk. Mucous plugging also improved.

**FIGURE 2:**
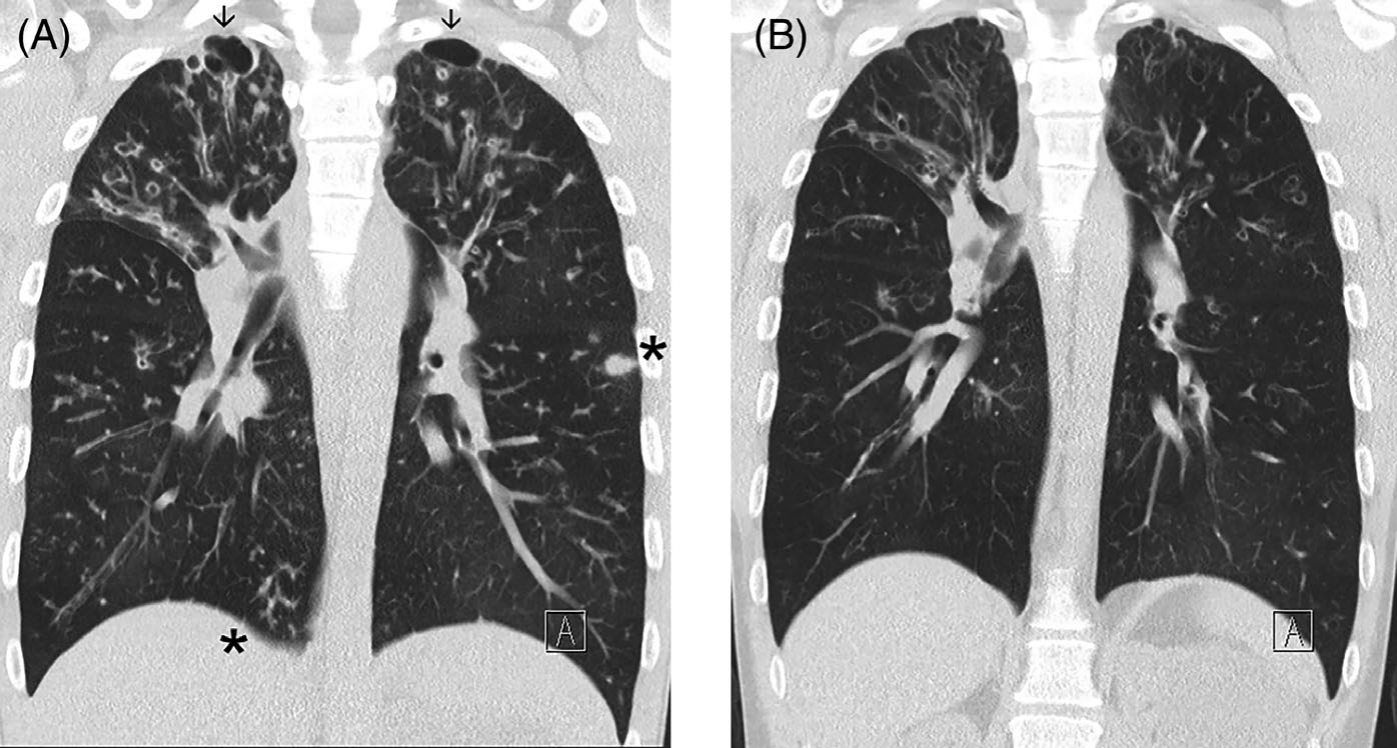
CT scans (A) before and (B) after elexacaftor + tezacaftor + ivacaftor for case 2

The second case, a 30‐year‐old male, had lung function of FEV_1_/FVC 0.98/2.09 (21%, 36% predicted) and required non‐invasive ventilation and supplemental oxygen whilst awaiting lung transplant. He commenced ETI and after 12 months his spirometry had increased to 1.32/2.93 (28%, 51% predicted). Non‐invasive ventilation and supplemental oxygen were ceased and the patient was taken off the lung transplant waiting list. CT scans pre and post ETI are shown in Figure 2A, B with reductions in the peripheral cysts at both apices (arrows) and mucous plugging (asterisks) as well as reduction in bronchial wall dilatation and thickening.

**FIGURE 3:**
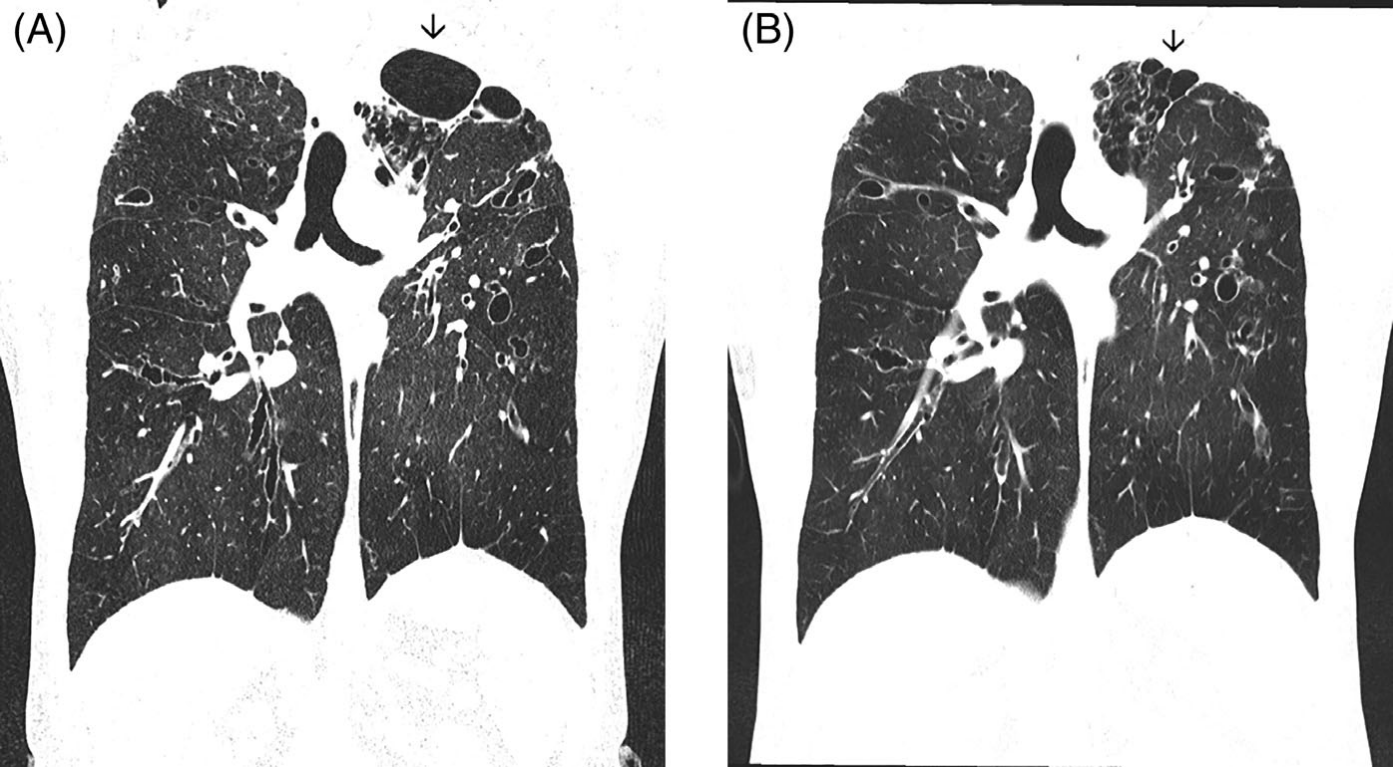
CT scans (A) before and (B) after elexacaftor + tezacaftor + ivacaftor for case 3

The third case, a 32‐year‐old female, with lung function of 0.89/2.20 (25%, 52% predicted) was initially commenced on dual therapy (tezacaftor‐ivacaftor; TI) but developed an acute rash. TI was ceased and the rash resolved. It was later re‐introduced with a rapid desensitization protocol and 3 days later she commenced ETI without further rash. After 14 months, her spirometry had increased to 1.46/3.08 (42%, 73% predicted). HRCT scans pre and post ETI are shown in Figure 3A, B demonstrating almost complete resolution of the cysts at the left apex (arrows) in addition to slight reduction in bronchial wall thickening.

## DISCUSSION

These three cases confirm the rapid and sustained improvements in lung function with elexacaftor + tezacaftor + ivacaftor (ETI) as shown previously.^5^ Whilst the improvement in mucous plugging and wall thickening were expected, the reduction in the structural changes of bronchiectasis is novel. In the past, resolution of bronchial wall dilatation in young children has been documented in the AREST CF cohort.^7^ In adults, occasional reports of reversible bronchial wall dilatation in the setting of dense consolidation have led to the term ‘pseudo‐bronchiectasis’.^8^, ^9^ This implies a transient dilatation of the airways due to the surrounding parenchymal changes, causing airway dilatation in keeping with cylindrical bronchiectasis. Furthermore, previous studies with ivacaftor for those with the G551D mutation have shown improvements in the Brody score of bronchiectasis severity, but did not document improvement in areas of varicose or cystic damage.^10^ Improvement in bronchial wall thickening and mucous plugging with ETI has been shown in studies using MRI^11^ and CT,^12^ but these studies did not show changes in the more severe structural abnormalities.

Complete or partial resolution of cystic bronchiectasis is an exciting new finding. The ‘vicious vortex’ of bronchiectasis suggests that once instigated, ongoing cycles of mucous retention, infection and inflammation result in airway and pulmonary destruction.^4^ In people with CF, the instigating factor of CFTR dysfunction can now be ameliorated with CFTR modulators. Reversal of bronchiectasis, even in the context of continued airway infection, suggests a more dynamic relationship between the instigating factor(s) causing bronchiectasis (e.g., CFTR dysfunction, as in these cases) and the ongoing airway changes as measured on HRCT.

With increasing recognition of the importance of both inflammation and infection in the maintenance and progression of the ‘vicious vortex’ of bronchiectasis,^4^ the exact factors involved in the vicious vortex of bronchiectasis need to be considered. Is CFTR dysfunction only an ‘instigating factor’ in the pathogenesis of bronchiectasis? Or does it have further role(s) in ongoing disease progression? In cigarette smokers, functional defects in CFTR have been described both locally^13^ and systemically,^14^ with recent work showing that CFTR modulators can improve both mucous transport and pathological changes of chronic bronchitis in a ferret model.^15^ Taken together these findings raise the possibility that the progression of airway disease may be related to downregulation of CFTR.

Further studies will be necessary to confirm these exciting preliminary findings, to quantify the improvements seen following CFTR modulation and to determine the mechanism(s) involved. The question remains whether increasing CFTR function will also improve bronchiectasis of other causes. This is an exciting time to be in the world of precision medicine as it relates to bronchiectasis.

## AUTHOR CONTRIBUTIONS

Peter Middleton Conceptualisation, collected clinical history and data, drafted initial manuscript, reviewed and revised the manuscript. Nicholas Simmonds Conceptualisation, collected clinical history and data, reviewed and revised the manuscript.

## CONFLICT OF INTEREST STATEMENT

Peter Middleton reports grants from Vertex Pharmaceuticals, during the conduct of the study; personal fees from Vertex Pharmaceuticals, outside the submitted work. Nicholas Simmonds reports personal fees from Vertex Pharmaceuticals, Chiesi, Gilead, Menarini, Zambon, outside the submitted work.

## ETHICS STATEMENT

The authors declare that appropriate written informed consent was obtained for the publication of this manuscript and accompanying images.

## Data Availability

The data that support the findings of this study are available on request from the corresponding author. The data are not publicly available due to privacy or ethical restrictions.
